# Interplay of miRNAs and Canonical Wnt Signaling Pathway in Hepatocellular Carcinoma

**DOI:** 10.3389/fphar.2018.00657

**Published:** 2018-06-21

**Authors:** Xiaobo Nie, Yiran Liu, Wei-Dong Chen, Yan-Dong Wang

**Affiliations:** ^1^Key Laboratory of Receptors-Mediated Gene Regulation and Drug Discovery, School of Medicine, Henan University, Kaifeng, China; ^2^Department of Pathology, Henan Provincial People’s Hospital, Zhengzhou, China; ^3^Key Laboratory of Molecular Pathology, School of Basic Medical Science, Inner Mongolia Medical University, Hohhot, China; ^4^State Key Laboratory of Chemical Resource Engineering, College of Life Science and Technology, Beijing University of Chemical Technology, Beijing, China

**Keywords:** HCC, miRNA, Wnt, hepatocellular carcinoma, β-catenin

## Abstract

Hepatocellular carcinoma is one of the leading causes of cancer death worldwide and the activation of canonical Wnt signaling pathway is universal in hepatocellular carcinoma patients. MicroRNAs are found to participate in the pathogenesis of hepatocellular carcinoma by activating or inhibiting components in the canonical Wnt signaling pathway. Meanwhile, transcriptional activation of microRNAs by canonical Wnt signaling pathway also contributes to the occurrence and progression of hepatocellular carcinoma. Pharmacological inhibition of hepatocellular carcinoma pathogenesis and other cancers by microRNAs are now in clinical trials despite the challenges of identifying efficient microRNAs candidates and safe delivery vehicles. The focus of this review is on the interplay mechanisms between microRNAs and canonical Wnt signaling pathway in hepatocellular carcinoma, and a deep understanding of the crosstalk will promote to develop a better management of this disease.

## Introduction

Hepatocellular carcinoma (HCC) is the fifth most common malignancy and the second leading cause of cancer death worldwide, with more than 750,000 new cases diagnosed and 700,000 cancer deaths occurred annually. Approximately more than 80% of HCC-related deaths can be attributed to the chronic viral hepatitis infection (hepatitis B or C), other risk factors including alcohol-related cirrhosis, non-alcoholic steatohepatitis, nitrosamine compounds or aflatoxin exposure, and algal toxins from contaminated water (Chuang et al., 1992; Ledda et al., 2017). The routine use of blood testing and image evaluation have obviously improved the detection of HCC, and improved living standard and childhood vaccination against hepatitis B have significantly reduced its incidence effectively in the past few decades (Blumberg et al., 1975). Unfortunately, the usual survival outcome of HCC is still poor, because it is often detected an advanced state or it is diagnosed after metastasis with no noticeable or unspecific symptom in the early stage. Hepatectomy and liver transplant are preferred therapies for HCC whereas they are limited to the liver function, and now a combination treatment including chemotherapy, radiation, interventional therapy and targeted therapy have been mostly applied and the survival rates of patients with HCC have been improved. However, there is no cure for HCC currently and it is still meaningful to explore detailed molecular mechanisms that contribute to improve diagnostic and therapeutic management of HCC.

Hepatocellular carcinoma is a complex polygenetic disease ascribed to the interactions between genetic predisposition and environmental factors. Inactivation or loss of tumor suppressor genes such as p21, p53, Rb and PTEN and activation of oncogenes including AKT and N-ras could induce the carcinogenesis of HCC (Martin and Dufour, 2008; Ho et al., 2012). Meanwhile, HCC is also strongly correlated with abnormality of cellular signal pathways, including EGFR, VEGFR, MAPK, IGFR, Wnt signaling pathways (Whittaker et al., 2010; Berasain et al., 2011). Among them Wnt signaling pathways attract significant attention because its role in the pathogenesis of colorectal cancer has become clear (Bienz and Clevers, 2000), and more researches demonstrated that changes of Wnt signaling may be the common pathogenetic basis of cancers. Although it has been found that Wnt signaling pathway is mainly involved in the occurrence and development of HCC by activating its downstream target genes (Thompson and Monga, 2007), the function of components of Wnt signaling pathway within the HCC context remains partially understood.

MicroRNAs (miRNAs) are a class of short (18–25 nucleotides) non-coding RNAs that repress target gene expression through the interaction with the 3′-untranslated region (3′-UTR) of their target genes, leading to either mRNA degradation or the inhibition of mRNA translation. Studies have shown that miRNAs play important roles in the development of cancer including HCC (Zhang et al., 2007; Klingenberg et al., 2017), through acting on the corresponding target mRNAs involved in cellular proliferation, differentiation, apoptosis and metastasis. Previous studies have identified a number of miRNAs participating in the pathogenesis of HCC by regulating components in Wnt signaling pathway. In the recent days, Rahmani et al. (2017) reported the roles of Wnt/β-catenin signaling pathway and related miRNAs in colorectal cancer. In this review, the crosstalk between miRNAs and Wnt signaling pathway in the pathogenesis of HCC is summarized.

## Canonical Wnt Signaling Pathway in HCC Pathogenesis

Aberrant canonical Wnt signaling pathway is closely associated with the progression and metastasis of various cancers, including HCC, and gene mutations or abnormal expression of Wnt signaling components cause activation of canonical Wnt signaling pathway in HCC (Lee et al., 2006). For example, in a specific subtype of familial and sporadic hepatocellular carcinomas, DICER1 mutations in liver carcinogenesis are associated with CTNNB1 (encoding β-catenin) mutations and lead to the β-catenin activation (Caruso et al., 2017). Dickkopf 2 (DKK2) is an antagonist of Wnt3a, the reduction of its function due to the loss of *DKK2* alleles leads to unchecked canonical Wnt signaling and contributes to HCC oncogenesis (Lin et al., 2016). In clinical HCC samples, CTNND1 (δ-catenin) expression was found to be up-regulated significantly in cancer tissues compared with paired normal liver tissues, and overexpression of CTNND1 in HCC cell lines promotes carcinous characters through indirectly enhancing Wnt/β-catenin signaling (Tang et al., 2016). Similarly, secreted frizzled-related protein-1 (SFRP1) is a well-known inhibitor of Wnt/β-catenin signaling and patients with lower SFRP1 expression level in tumor tissue have poor overall survival rate in HCC (Davaadorj et al., 2016). In addition, Wnt/β-catenin can be activated by epigenetic modifications, such as lncRNA or miRNA regulation, this review will focus on the interplay between canonical Wnt signaling and miRNAs in later sections, and we hope it will facilitate the development of improved therapies for HCC.

## miRNAs Targeting Wnt Ligands/Receptors and Associated Inhibitory Proteins

Wnt ligands are secreted as lipid-modified signaling glycoproteins comprising 19 family members in human being, and canonical Wnt signaling pathway is originally activated by the binding of Wnt ligands to its receptor such as FZD and LRP5 or LRP6. The pathway transduction will be interrupted if miRNAs target any of these Wnt ligands or receptors. miR-122 expression level is found to be decreased significantly in human HCC tissue samples and cell lines, and overexpression of miR-122 inhibits proliferation but promotes hepatoma cell apoptosis by repressing Wnt1 expression, subsequently leads to blocking Wnt1/β-catenin/TCF signaling pathway (Xu J. et al., 2012; Ahsani et al., 2017) (**Table 1**). Meanwhile, Wnt1 is also targeted by endogenous miR-148a in HCC cells. Yan et al. (2014) showed that miR-148a expression level in metastatic HCC tissues is lower than that of nonmetastatic ones, and overexpression of miR-148a blocks the metastasis of HCC cells by suppressing the epithelial-mesenchymal transition (EMT) and acquisition of cancer stem cells (CSCs)-like properties through affecting the canonical Wnt signaling pathway. Furthermore, miR-148b is confirmed as another miRNA regulating Wnt1. It is downregulated in human HCC tissues. Patients with higher miR-148b expression in tumor tissues are shown to have a better prognosis, therefore miR-148b functions as a tumor suppressor in HCC through targeting WNT1/β-catenin pathway (Zhang J.G. et al., 2015) (**Table 1**). However, other Wnt family members have not been reported to be regulated directly by miRNAs in HCC.

**Table 1:** Oncogenic and tumor suppressor miRNAs targeting the components of canonical Wnt signaling pathways in the pathogenesis of HCC.

miRNAs	Targets in canonical Wnt signaling	Effect	Reference
**Targeting Wnt ligands/receptors and associated proteins**			
miR-122	Wnt1	Tumor suppressor	Xu J. et al., 2012; Ahsani et al., 2017
miR-148a	Wnt1	Tumor suppressor	Yan et al., 2014
miR-148b	Wnt1	Tumor suppressor	Zhang J.G. et al., 2015
miR-152	Wnt1	Tumor suppressor	Huang et al., 2014
miR-27a	FZD7	Tumor suppressor	Chen Z. et al., 2013
miR-199a	FZD7	Tumor suppressor	Song et al., 2014
miR-1269a	LRP6	Tumor suppressor	Min et al., 2017
miR-202	LRP6	Tumor suppressor	Zhang et al., 2014
miR-432	LRP6	Tumor suppressor	Jiang et al., 2015
miR-126-3p	LRP6	Tumor suppressor	Du et al., 2014
miR-610	LRP6	Tumor suppressor	Zeng et al., 2014
miR-181a	WIF-1	Oncogene	Ji et al., 2014
miR-522	DKK1, sFRP2	Oncogene	Zhang H. et al., 2016
miR-200b	BMI1	Tumor suppressor	Wu W.R. et al., 2016
miR-218	BMI1	Tumor suppressor	Fu et al., 2015
**Targeting β-catenin**			
miR-214	β-catenin	Tumor suppressor	Wang et al., 2012; Xia et al., 2012
miR-200a	β-catenin	Tumor suppressor	Liu et al., 2013
miR-320a	β-catenin	Tumor suppressor	Lu et al., 2017
miR-338-3p	β-catenin	Tumor suppressor	Zhang T. et al., 2016
miR-33a	β-catenin	Tumor suppressor	Fang et al., 2013
miR-153	WWOX (β-catenin inhibitor)	Oncogene	Hua et al., 2015

**miRNAs**	**Target in Wnt signaling**	**Effect**	**Reference**
**Targeting β-catenin interacting proteins**			

miR-9	E-cadherin (CDH1)	Oncogene	Tan et al., 2010; Drakaki et al., 2015
miR-106b	APC	Oncogene	Shen et al., 2013
miR-155	APC	Oncogene,	Zhang et al., 2012
miR-107	AXIN2	Oncogene	Zhang J.J. et al., 2015
miR-1246	AXIN2, GSK3β	Oncogene	Chai et al., 2016
miR-920	β-TRCP	Tumor suppressor	Chen et al., 2010
**Targeting transcription factors in canonical Wnt pathway**			
miR-139	TCF-4	Tumor suppressor	Gu et al., 2014
miR-30-5p	BCL9	tumor suppressor	Zhao et al., 2014
miR-101	NLK2	Tumor suppressor	Shen et al., 2014
miR-181	NLK	Oncogene	Ji et al., 2009
miR-20a-5p	RUNX3	Oncogene	Chen et al., 2016
miR-130a	RUNX3	Oncogene	Xu N. et al., 2012
miR-129-2	SOX4	Tumor suppressor	Chen X. et al., 2013
miR-935, miR-24, miR-184	SOX7	Oncogene	Ma et al., 2014; Wu et al., 2014; Liu et al., 2017
miR-221	HDAC6	Oncogene	Bae et al., 2015
**targeting β-catenin responsive genes**			
miR-29b	MMP-2	Tumor suppressor	Fang et al., 2011
miR-451	MMP-9	Tumor suppressor	Arii et al., 1996
miR-199a	MMP-9	Tumor suppressor	Zhang et al., 2013
miR-451	c-Myc	Tumor suppressor	Huang et al., 2015
miR-34a	c-Myc	Tumor suppressor	Xu et al., 2015
let-7a	c-Myc	Tumor suppressor	Deng et al., 2016
miR-199a-3p	CD44	Tumor suppressor	Henry et al., 2010
miR-101	COX2	Tumor suppressor	Zheng et al., 2015
miR-19a	cyclin D1	Tumor suppressor	Zhang Y. et al., 2015
miR-20a	cyclin D1	Tumor suppressor	Karimkhanloo et al., 2017
miR-384	IRS1- cyclin D1	Tumor suppressor	Lai et al., 2016

**FIGURE 1:**
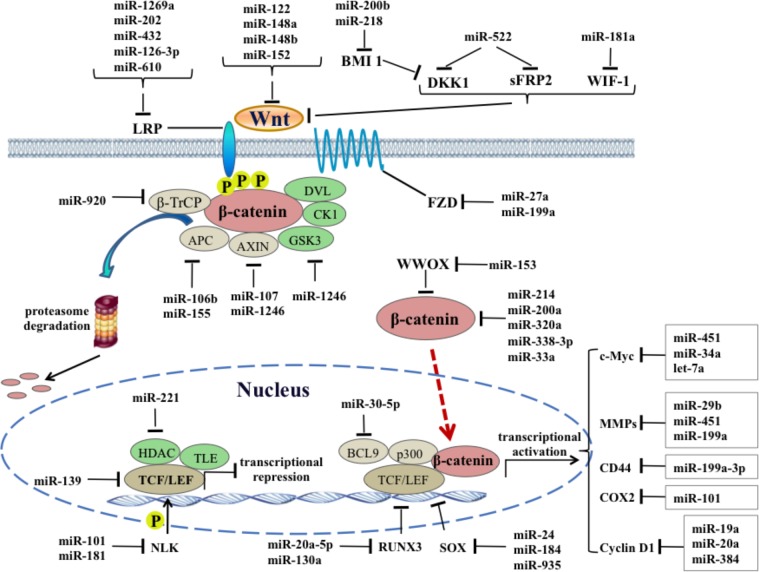
Regulatory functions of oncogenic and tumor suppressor miRNAs on canonical Wnt signaling pathways in HCC.

Another group of miRNAs regulating Wnt receptors also act like tumor suppressors. miR-199a is frequently downregulated in HCC cells and tissues, and lower miR-199a expression in HCC tissues indicates malignant potential and poor prognosis. Restoration of miR-199a in HCC cells could inhibit cell proliferation and survival by targeting FZD7 and its downstream genes, including β-catenin, cyclin D1 and c-Myc (Song et al., 2014). Similarly, miR-27a expression is reported to be downregulated in the multidrug-resistant HCC cell line, and upregulation of miR-27a could enhance the sensitivity of HCC cells to chemotherapy drugs through targeting the FZD7/β-catenin pathway (Chen Z. et al., 2013). Other miRNAs including miR-1269a, -202, -432, -126-3p and 610 could directly target LRP5 to inhibit the canonical Wnt signaling pathway. For example, miR-202 and miR-126-3p were proved to suppress cell proliferation, metastasis and angiogenesis of HCC by downregulating LRP6 expression (Du et al., 2014; Zhang et al., 2014). Inhibiting miR-610 and miR-432 enhances proliferation and tumorigenicity of HCC cells through directly suppressing multiple regulators of the Wnt/β-catenin signaling cascade, including LRP6 (Zeng et al., 2014; Jiang et al., 2015). Interestingly, a single nucleotide variant in miR-1269a could reduce its own anticancer effect and even promote the occurrence and process of HCC by disinhibiting the oncogene LRP6 (Min et al., 2017) (**Figure 1**).

Conversely, miRNAs targeting the inhibitory factors of Wnt ligands will be oncogenic and activates Wnt/β-catenin pathway. As a secreted lipid binding protein that binds to Wnt proteins and inhibits Wnt signaling pathway, Wnt inhibitory factor-1 (WIF-1) is identified as a direct and functional target of miR-181a in colorectal cancer, and an ectopic expression of miR-181a promotes tumor growth and liver metastasis (Ji et al., 2014). It can be speculated that WIF-1 could also be targeted by miR-181a due to the extensive low expression of WIF-1 and an opposite tendency of miR-181a in HCC (Deng et al., 2010). Dickkopf-1 (DKK1) and secreted frizzled-related protein 2 (SFRP2) are both antagonists of Wnt signaling. Sun’s group has reported that miR-522 promotes cell proliferation of HCC by targeting DKK1 and SFRP2 and activating Wnt signaling (Zhang H. et al., 2016), whereas other researchers report that DDK1 is overexpressed in HCC cells and tissues, and promotes HCC cell migration and invasion through β-catenin/MMP7 pathway (Chen L. et al., 2013). As an activator of the Wnt pathway by repressing DKK family, polycomb group protein BMI1 often overexpresses in HCC and is required for self-renewal of HCC CSC. In particular, BMI1 mediates activation of Wnt signaling, leading to a further transcriptional autoactivation of itself. Forced expression of miRNA-200b in HCC cells dramatically represses malignant features including cell proliferation, colony formation, and invasion by targeting BMI1 (Wu W.R. et al., 2016). Moreover, miR-218 is a tumor suppressor with an inhibitory effect on BMI1 expression in HCC pathology (Fu et al., 2015). Thus, any miRNAs that can regulate Wnt ligands/receptors and associate inhibitory proteins directly or indirectly could influence the transduction of the canonical Wnt signaling pathway.

## miRNAs Targeting Transcription Factors in Canonical Wnt Pathway

TCF/LEF transcription factors mediate a response to Wnt signals by binding to the stabilized β-catenin in nucleus, resulting in activation of Wnt-responsive genes. Negative regulation at the protein level of TCF/LEF transcription factors and associated coactivators/co-repressors by miRNAs will affect the signal transduction of Wnt/β-catenin pathway and pathogenesis of HCC. TCF-4 promotes initiation and progression of cancers when binding to the β-catenin. However, in the absence of β-catenin, it will become a repressor of the transcription of β-catenin target genes by binding to co-repressors. In the previous study, a significant downregulation of miR-139 expression was observed in clinical HCC tissues, and miR-139 functions as an anticancer gene and suppresses the β-catenin/TCF-4 transcriptional activity by targeting TCF-4 (Gu et al., 2014). BCL9 is a coactivator for TCF/LEF transcription factor and targeted by miR-30-5p, suppressing cancer progression in multiple myeloma cancers (Zhao et al., 2014), whereas its upstream miRNAs have not yet been found in HCC. As an evolutionarily conserved protein kinase involved in canonical Wnt signaling, nemo-like kinase (NLK) positively regulates Wnt/β-catenin signalling by phosphorylating LEF1 and induces its dissociation from histone deacetylase, thereby allowing transcription activation (Ota et al., 2012). NLK2 is significantly overexpressed in HCC tissues and miR-101 could inhibit multiple malignant phenotypes of HCC cells through regulating abnormal NLK2 activity (Shen et al., 2014). In contrast, NLK also appears to function as a tumor suppressor in HCC because a study has reported that miR-181 induces HCC cell quantity and tumor initiating ability through direct inhibition of NLK mRNA translation (Ji et al., 2009).

## miRNAs Targeting β-Catenin Responsive Genes in Canonical Wnt Pathway

The regulation effect of canonical Wnt signaling pathway on tumorigenesis is achieved by changing the expression levels of diversified oncogenes that exhibit tissue-specific expression. So far, more than five hundred Wnt target genes have been identified by the gene chip and the whole genome ChIP-sequencing, forming a rich Wnt target genome. MMPs, c-Myc, CD44, cyclin D1, and COX2, etc., are main target genes of canonical Wnt pathway in HCC and their roles have been intensively studied in the past decades. As the main enzymes that degrade extracellular matrix in the body, MMPs facilitate the invasion and metastasis of various malignant tumors including HCC. Overexpression of MMP-2 has been observed in patients with chronic hepatic disease and HCC tissues and a significant correlation exists between MMP-2 level and liver function (Kuyvenhoven et al., 2003). Zhuang et al. found that miR-29b suppresses tumor angiogenesis, venous invasion and metastasis, at least partially, by directly targeting MMP-2 expression in HCC (Fang et al., 2011). Similarly, the expression level of MMP-9 could be regarded as an indicator to detect the recurrence, invasion and metastasis of primary HCC, and overexpression of miR-451 abrogates human hepatoma cell growth and invasion, accompanied with the decrease of MMP-9 (Arii et al., 1996). Propofol, a commonly used intravenous anesthetics, was found to inhibit the invasiveness of HCC cells by elevating miR-199a expression and subsequent downregulating MMP-9 expression (Zhang et al., 2013) (**Figure 1**).

c-Myc is a transcription factor that binds DNA in a non-specific manner and regulates cell cycle positively. A recent study uncovered that the oncogene c-Myc is identified as a direct target of miR-451 and miR-451 downregulation-induced c-Myc overexpression leads to acquisition of EMT phenotype in HCC cells (Huang et al., 2015). In another HCC cell line, miR-34a induces cellular senescence via inhibiting the telomerase activity, at least partially by targeting c-Myc (Xu et al., 2015). CD44 is described as a CSC marker in HCC and its expression is induced by canonical Wnt signaling, resulting in the recurrence and increased metastatic ability of HCC. miR-199a-3p was shown to reduce proliferation of CD44-positive HCC cell lines and sensitize the cells to chemotherapy drugs by repression of CD44 (Henry et al., 2010). COX-2 is highly expressed in well-differentiated HCC and can promote tumor cell growth and neovascularization through arresting cell cycle and blocking apoptosis. A recent study has shown that systemic delivery of lentivirus-mediated miR-101 strongly abrogated HCC tumorigenesis in the liver and intrahepatic and distant metastasis *in vivo*, by downregulating several targets including COX-2 (Zheng et al., 2015). Cycin D1 is a typical target gene in canonical Wnt pathway, its overexpression could accelerate the G1/S phase transition of cell cycle and cause uncontrolled cell proliferation. Recently, several studies revealed potential links between miRNAs and cyclin D1 in HCC. For example, a systematic investigation based on miRNA-mediated gene regulatory network revealed that miR-19a plays an inhibitory role in HCC via suppressing cyclin D1 expression, indicating therapeutics targeting miR-19a/cyclin D1 axis might be benefit for HCC (Zhang Y. et al., 2015). Moreover, bioinformatics prediction and experimental validation also proved that miR-20a could be another new therapeutic agent for HCC through targeting cyclin D1 (Karimkhanloo et al., 2017) (**Figure 1**).

## miRNAs Targeted by Canonical Wnt Signaling Pathway in HCC

Presently, more researches focus on the identification of novel miRNAs and their responsive targets in cancer, whereas the mechanisms by which miRNAs are activated or inhibited are obscure. It is interesting to find that components of canonical Wnt signaling pathway could conversely regulate miRNAs in HCC (**Table 2**). For instant, Wu *et al.* demonstrated that a regulatory feedback loop exists between miR-17-5p and c-Myc, in which miR-17-5p could inhibit metastasis and invasion of HCC cells by suppressing c-Myc, and miR-17-5p, in turn, is induced by activated c-Myc as a transcription factor, although detailed mechanism is still needed to be elucidated (Liu et al., 2016). Moreover, miR-101 is a direct target gene epigenetically silenced by c-Myc in HCC cells and overexpression of c-Myc in HCC samples was closely related to lower miR-101 levels and poorer prognosis of HCC patients (Wang et al., 2014). Similarly, c-Myc is pathologically activated in HCC and induces hepatocarcinogenesis through a novel miRNA-mediated feedback loop comprised of miR-148a-5p and miR-363-3p (Han et al., 2013). In addition to upregulating target genes in canonical Wnt signaling pathway, β-catenin/TCF4 complex could also activate the transcription of miRNAs and produce a positive feedback regulatory loop in HCC. Ji et al. (2011) reported that several putative β-catenin/TCF4 binding sites are identified in the promoter region of the miRNA-181a-2 and miRNA-181b-2 transcripts, and four members in miRNA-181 family are positively associated with β-catenin expression in HCC. This is in line with the previous report which demonstrates that miR-183/96/182 cluster is activated by Wnt/β-catenin/TCF3 signaling in HCC and promotes cell migration and invasion (Leung et al., 2015). Besides, Wnt/β-catenin signaling is found to act on the transcription of miRNA-770, subsequently exerting a positive influence on the tumorigenesis of HCC.

**Table 2:** miRNAs regulated by canonical Wnt signaling pathways in the pathogenesis of HCC.

miRNAs	Targeted by canonical	Effect of miRNAs	Reference
**Wnt signaling**			
miR-17-5p, miR-101	c-Myc	Tumor suppressor	Wang et al., 2014; Liu et al., 2016
miR-148a-5p, miR-363-3p	c-Myc	Tumor suppressor	Han et al., 2013
miR-181	β-catenin/TCF4	Oncogene	Ji et al., 2011
miR-183/96/182	β-catenin/TCF3	Oncogene	Leung et al., 2015
miR-770	Wnt/β-catenin	Oncogene	Wu W.J. et al., 2016
miR-122	GSK-3β	Tumor suppressor	Zeng et al., 2010
miR-34a	β-catenin	Oncogene	Gougelet et al., 2016

## Pharmacological Inhibition of HCC Pathogenesis by miRNAs

The complicated and sophisticated regulation on abnormally expressed target genes during tumorigenesis makes miRNAs promising therapeutic agents or targets for cancer therapy. Functional studies indicated that downregulated tumor suppressor miRNAs could be restored to treat cancers by using miRNA mimics, and variety of antimiRs in the form of miRNA sponges, anti-miR oligonucleotides and small molecule inhibitors could be developed to inhibit or compete with oncogenic miRNA in preclinical development. Recently, several miRNA-targeted therapeutics have reached preclinical or clinical development for cancer therapy including HCC (Rupaimoole and Slack, 2017). MiR-155 is present at abnormally high levels in HCC and several other cancers, as a locked nucleic acid (LNA) to antagonize miR-155, MRG-106 is used for patients with certain lymphomas and leukemias and the ongoing Phase I clinical trial (ClinicalTrials.gov identifier: NCT02580552) which is available for assessing its safety, tolerability and pharmacokinetics will be completed in December 2018, thus the potential inhibition effect of MRG-106 on canonical Wnt signaling pathway will make it an excellent candidate for HCC therapy.

Chu et al. (2017) reported that miR-1247-5p has the potential to be a tumor suppressor in HCC by targeting Wnt3. It also has been reported that miR-337 regulates the Wnt/β-catenin signaling pathway to inhibit HCC progression by targeting high-mobility group AT-hook 2 (Cui et al., 2018). miR-221 is one of the most significantly upregulated miRNAs in solid tumors such as HCC, pancreas and lung cancers. It targets the tumor suppressors such as PTEN, metalloproteinase inhibitor 3 (TIMP3) and HDAC6 in canonical Wnt signaling (Garofalo et al., 2009; Bae et al., 2015). Intravenously delivering to a valid orthotopic mouse model of HCC blocks tumor cell proliferation and elevates mouse survival. Furthermore, a cholesterol-modified form of anti-miR-221 exhibits improved liver tissue distribution and pharmacokinetics than unmodified oligonucleotide, suggesting a broad application prospects for patients with HCC (Park et al., 2011). These reports support the potentiality for miRNAs as targets for treatment of HCC in the future.

At present, several main challenges are still blocking the movement of miRNAs therapeutics into clinically development, including the identification of the best miRNAs or targets for specific cancer type, off-target effect due to the similar sequence of miRNAs within the same family, severe toxicity and immunogenicity of chemically modified miRNAs, low bioavailability and delivery efficiency of miRNA delivery vehicles. Additionally, one miRNA can regulate serial different genes and its inhibition can affect undesirably other biological pathways. However, with the advances in biotechnology and great efforts of scientists, miRNA-based therapeutics are still promising to become the clinical reality for cancer treatment.

## Conclusion

It is well known that oncogenic or tumor suppressor miRNAs modulates the HCC cell proliferation, invasion, metastasis and drug susceptibility, through targeting components or regulatory factors in canonical Wnt signaling pathway. Conversely, feedback regulation of miRNAs by canonical Wnt signaling pathway could also contribute to the pathogenesis of HCC. In this review, we summarize the recent findings on the interaction between miRNAs and canonical Wnt signaling pathway in HCC, and it remains to be deeply investigated the specific upstream regulators for the miRNAs that target this pathway. But all in all, miRNA-based therapeutics for HCC and other cancers are now in clinical trials despite plenty of technical challenges, and increased knowledge about the interplay between miRNAs and canonical Wnt signaling pathway will reveal the underlying mechanisms of HCC and contribute to novel miRNAs-based therapeutic designs.

## Author Contributions

XN wrote the manuscript. YL edited the manuscript. W-DC and Y-DW edited and modified the manuscript.

## Conflict of Interest Statement

The authors declare that the research was conducted in the absence of any commercial or financial relationships that could be construed as a potential conflict of interest.
